# Evaluation of Prevention Programs for Grandparent Caregivers: a Systematic Review

**DOI:** 10.1007/s11121-023-01594-2

**Published:** 2023-10-14

**Authors:** Athena Chung Yin Chan, Timothy F. Piehler

**Affiliations:** 1 Department of Human Development and Family Sciences, Texas Tech University, P.O. Box 41230, Lubbock, TX 79409 USA; 2 Deparment of Family Social Science, University of Minnesota, 290 McNeal Hall, 1985 Buford Avenue, Saint Paul, MN 55108 USA

**Keywords:** Grandparent caregivers, Core components, Prevention programs, Program evaluation

## Abstract

This systematic review critically evaluates 21 grandparent-focused prevention programs designed to benefit grandparent caregivers or target grandparent caregivers as agents of change for grandchild development. These grandparent programs were published in 35 peer-reviewed articles in the last 31 years. For each program, we described the phase of development of program (i.e., feasibility, pilot, and efficacy), delivery characteristics, and adaptations of existing evidence-based programs to meet the needs of grandparent caregivers in diverse sociocultural backgrounds. For programs tested under a controlled trial design, we evaluated core components and preliminary effects on targeted outcomes. Most studies targeted grandparents who served as primary or custodial caregivers in the USA, while limited studies targeted supplementary grandparent caregivers. Thirteen programs which were evaluated using pretest–posttest designs demonstrated feasibility and acceptability of the grandparent programs. Moreover, eight programs which were evaluated using randomized-controlled trials (RCTs; 5 pilot and 3 full-scale efficacy trials) demonstrated promising outcomes. Additionally, we identified six core components and 17 specific elements in eight programs evaluated using RCT designs. Overall, we provide a comprehensive critique of this body of literature as well as directions for future studies, including the need to develop programing tailored to grandparent caregivers with unique needs.

**Supplementary Information:**

The online version contains supplementary material available at 10.1007/s11121-023-01594-2.

Grandparents can be key family resources in providing care for and positively influencing the development of grandchildren (Stelle et al., 2010). Across diverse family structures and cultural contexts, grandparents play different family roles and vary in the extent of their involvement in grandchild care globally (Shwalb & Hossain, 2017). The spectrum of grandparent roles ranges from occasional interactions with grandchildren (i.e., informal caregivers) to regular provision of childcare for grandchildren (i.e., supplementary, primary, and custodial caregivers). This systematic review focuses on prevention programs targeting grandparent caregivers with regular provision of childcare. Custodial or primary caregivers are grandparents who have sole responsibility for taking care of their grandchildren with an absence of adult children in skipped-generation household, whereas supplementary caregivers are grandparents who co-reside with their adult children and collaboratively care for their grandchildren as a team in multigenerational households (Dunifon et al., 2014; Hayslip et al., 2019; Kim et al., 2017). The social interaction learning (SIL) model states that social interaction in the family context shapes behavior. Accordingly, family caregivers (e.g., parents) are the primary socializers and treatment agents for change in preventions programs for their children (Forgatch & Martinez, 1999). Thus, in the current systematic review, we posit that grandparent caregivers can serve as agents of change to promote healthy development and prevent adjustment problems in grandchildren in diverse family structures.

Grandparent caregivers may face a number of challenges, including coping with aging, other transitions such as retirement, and associated financial concerns. Despite parenting the second time, grandparents, particularly primary or custodial caregivers, may experience role ambiguity in dual roles of being a caring grandparent and a surrogate parent (Dolbin-MacNab, 2006). Grandparent caregivers may need to acquire different parenting knowledge and skills for childcare of a new generation in the digital age (Kirby & Sanders, 2014a). Furthermore, an excessive caregiving burden from parenting demands may put grandparent caregivers’ physical and psychological well-being at risk (Chan et al., 2023). Moreover, grandparent caregivers may face various difficulties in navigating the complex relationships between adult children and grandchildren irrespective of whether their adult children are present or absent in the households (Hoang et al., 2022). Given the potential differential impacts of the caregiving role on grandparents, it is important to investigate whether and how prevention programs may support grandparent caregivers as the agents of change for grandchild development. Notably, there has been relatively limited attention in the prevention literature focused on supporting grandparents as agents of change relative to parents as primary caregivers.

We define grandparent-focused preventive programs (i.e., grandparent programs) as programs that benefit grandparent caregivers or target grandparent caregivers as agents of change in order to support grandchildren outcomes. The development of evidence-based grandparent programs is an emerging area, and notably lags behind the implementation and dissemination of evidence-based parenting programs grounded in the SIL model for decades (Forgatch & Martinez, 1999). To better understand the mechanisms of grandparent programs, we apply the SIL model to conceptualize the proximal and distal outcomes of grandparent programs in Fig. 1**.** Grandparent programs target grandparent caregivers, both primary/custodial caregivers and supplementary caregivers, as agents of change (i.e., proximal outcomes) for the adjustment of grandchildren living in both skipped generation and multigenerational households (i.e., distal outcomes). While grandparent caregivers are the targets of grandparent programs, grandchildren may or may not be directly involved in grandparent programs. Specifically, the primary aims of grandparent programs target the proximal outcomes of grandparent caregivers, including promoting the well-being of grandparent caregivers, supporting adaptive parenting practices (i.e., increasing positive and decreasing coercive parenting behavior), and enhancing skills to manage intergenerational family relationships. Through changing the behavior of the socializers (i.e., grandparent caregivers), the secondary aims of grandparent programs target distal outcomes in grandchildren, including promoting healthy development and preventing adjustment problems (e.g., reducing disruptive behavior).

**Fig. 1:**
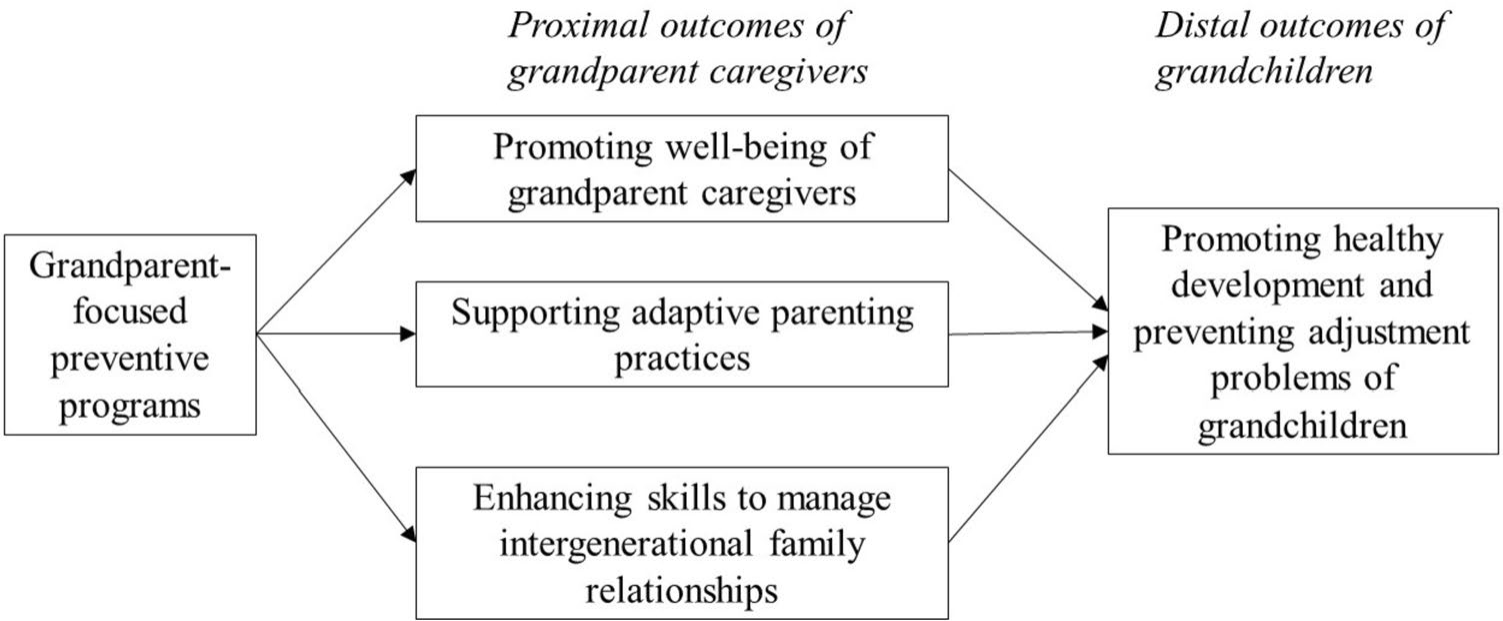
Conceptual model of grandparent-focused preventive programs targeting grandparent caregivers as agents of change. *Note*: This conceptual model illustrates how grandparent-focused preventive programs (grandparent programs) target grandparent caregivers as agents of change in order to promote healthy development and prevent adjustment problems of grandchildren. The primary aims of grandparent programs target the proximal outcomes of grandparent caregivers, while the secondary aims of grandparent programs target distal outcomes of grandchildren

## Existing Prevention Programs for Grandparent Caregivers

The existing development and evaluation of grandparent programs includes several limitations based on previous systematic reviews and meta-analysis (Chan et al., 2019; McLaughlin et al., 2017; Sherr et al., 2018; Sumo et al., 2018). First, many existing grandparent programs have only been evaluated in feasibility studies or pilot RCTs (Chan et al., 2019; Sherr et al., 2018). It is methodologically unsound to determine the efficacy or effectiveness of this programming with pilot RCT studies involving small sample sizes and potentially biased estimates (Kistin & Silverstein, 2015). To move the field forward in developing evidence-based programs, it is important to understand the phase of development in existing prevention programs for grandparent caregivers and evaluate the state of evidence for programs accordingly (Gitlin & Czaja, 2015). Second, most existing prevention programs target exclusively custodial/primary grandparents in the USA (Chan et al., 2019). We lack understanding of the range and scope of grandparent programs targeting supplementary caregivers and caregivers of diverse backgrounds, including the extent of attention to relevant cultural and contextual factors. For example, American grandparents from ethnic minority groups (e.g., Native American, Black, and Hispanic) are disproportionately more likely to be raising grandchildren in their middle adulthood (Hadfield, 2014). Moreover, few grandparent programs have been implemented outside the USA. Less is known about the extent to which existing grandparent programs were adapted to fit into the cultural values of grandparent caregivers of color or from countries outside of the USA. Third, we know little about how grandparent programs may be differentiated from prevention interventions for parent caregivers and kinship foster caregivers (Wu et al., 2020). It is unclear whether and to what extent we can adapt existing evidence-based parent programs to fit the needs of grandparent caregivers. Relatedly, the field also lacks a common set of evidence-based practices that benefit grandparent caregivers or target grandparent caregivers as the agents of change in supporting the development of grandchildren.

## Purpose of Systematic Review

By addressing these knowledge gaps, this review sought to better solidify our current understanding of the state of grandparent programming, with a goal of informing and advancing the development and evaluation of programming in this area. We sought to provide an in-depth evaluation of the wide range of prevention programs for grandparent caregivers according to the phase of development of the program (Gitlin & Czaja, 2015). Our review included a systematic examination of methodologies and preliminary outcomes of existing prevention programs targeting grandparent caregivers. We included all studies adopting randomized-controlled trials (RCTs), quasi-experimental, or pretest–posttest designs involving different phases of development of programs. There was no limit on the sample size of the programs. Moreover, we included both quantitative and qualitative evaluations. Furthermore, we sought to offer critiques and directions for future development of prevention programs for grandparent caregivers. Specifically, five objectives were addressed: (a) To identify the study design and developmental phase of studies used to evaluate grandparent programs, including programs evaluated in individual or multiple studies; (b) to identify delivery characteristics (i.e., program content, dosage and duration, modality, setting, and target caregivers) for grandparent programs; (c) to examine the extent to which existing grandparent programming has been adapted from other evidence-based programs (i.e., characteristics of populations and settings targeted for program adaptation, reasons and types of modifications, adaptation steps, and evaluation outcomes); (d) to evaluate the feasibility and preliminary outcomes of prevention programs according to their developmental phases; and (e) to identify the core components of RCT programs (i.e., pilot or efficacy).

## Methods

### Search Strategies

This systematic review followed the best practice recommendations for research syntheses and methodological steps for systematic reviews in the health sciences (Johnson & Hennessy, 2019). The parameters for the review were defined by elements captured by the PICOT and TOPICS acronyms (Johnson & Hennessy, 2019). The population (P) of interest were families involving grandparent caregivers in supporting and caring for grandchildren. The intervention (I) was the prevention programming designed to benefit grandparent caregivers or target grandparent caregivers as agents of change for the development of grandchildren. The comparison (C) utilized baseline levels of a condition to examine how much the condition changed over time and/or a control group receiving treatment as usual or alternative treatment (if applicable). The proximal outcomes (O) were grandparent caregivers’ parenting skills and well-being, as well as intergenerational family relationships. The distal outcome was grandchildren’s health and development. The period of time (T) included immediate intervention effects and longer-term outcomes. The study design (S) was limited to RCTs, quasi-experimental, or pretest–posttest designs.

Four behavioral, psychological, and biomedical databases, including PsycINFO via Ovid, MEDINE via Ovid, CINAHL, and Scopus, were selected. We conducted an initial search on May 22, 2020. Then, we evaluated the scope and quality of the literature to finalize the review protocol. We conducted a systematic review search on June 22, 2020, and updated on January 30, 2022, using a combination of keywords and controlled vocabulary for the following terms: (a) “grandparent caregivers,” “custodial grandparents,” “grandparents raising grandchildren”; (b) “intervention,” “training,” “program,” and “support group.” The search strategy is included as Appendix 1. We imported citations from all databases into an EndNote 20 library. The duplicate citations were removed within EndNote for abstract and full-text screening.

### Inclusion Criteria and Screening Eligibility

Inclusion criteria were (a) peer-reviewed articles; (b) written in English; (c) published after the year 1990 (which coincided with an increasing focus on understanding and supporting aging as encouraged by the United Nations; The United Nations General Assembly, 1991); (d) prevention programs adopting a RCT, quasi-experimental, or pretest–posttest design; (e) fully quantitative, fully qualitative, or mixed evaluation methods; (f) targeting the well-being of grandparent caregivers or using grandparent caregivers as the agents of change for the development of grandchildren; and (g) reporting preliminary outcomes of grandparenting, the well-being of grandparent caregivers and grandchildren, and/or family relationships. Notably, we included multiple studies evaluating different relevant outcomes of the same program targeting the same grandparent samples. Exclusion criteria were (a) program descriptions without evaluation of outcomes, (b) programs targeting kinship caregivers other than grandparents (e.g., aunts, siblings, etc.), and (c) a primary focus on grandchildren without involving grandparent caregivers in programming.

Following the Preferred Reported Items for Systematic Review and Meta-analysis (PRISMA) guidelines (Moher et al., 2009), a thorough search resulted in 1483 articles after removing duplicate citations. Figure S1 in the supplement displays the PRISMA flow chart for the article selection procedures. The intervention studies targeting grandparent caregivers as the agents of change for grandchild development were subsequently screened in two steps (i.e., 1407 articles were excluded under title/abstract, and 41 articles were excluded under full-text review). Using forward and backward searches, 35 articles met all the inclusion criteria (identified by * in the reference list).

We assessed the methodological quality and risk of bias of articles using (a) the quality assessment of controlled intervention studies tool (14 items) and (b) the quality assessment tool for before-after (pre-post) studies with no control group (12 items), both developed by the National Heart, Lung, and Blood Institute (2014). The tools were designed to critically appraise the validity of the study designs and evaluate potential biases in study methods (see Appendix 2). A final percentage was calculated for the number of fulfilled criteria (see Supplementary Table S1). Notably, studies were not excluded from this systematic review due to relatively low quality but were addressed in the discussion.

### Analytic Strategies

To understand the scope of studies and grandparent programs, we began the review by providing a broad overview, i.e., purposes, intervention characteristics, and sample characteristics. This was followed by an in-depth analysis to address each objective. The study design (Objective A) included RCT, quasi-experimental, or pretest–posttest designs. We categorized the developmental phase of programs (Objective A) as *Phase 1: Feasibility, proof of concept* (i.e., identifying an appropriate theoretical base for a program; refining program components, program contents, and delivery characteristics; and determining their acceptability, feasibility, and safety in any research designs); *Phase 2: Pilot testing, initial comparison with a control group* (i.e., identifying or refining appropriate outcomes and their measurement in evaluating the sensitivity of expected changes, determining the type of control group, evaluating potential treatment effects; and monitoring feasibility, acceptability, and safety of programs); *Phase 3: Full-scale efficacy trial* (i.e., enhancing the internal validity and demonstrating outcome efficacy with an appropriate alternative); and *Phase 4: Effectiveness trial* (i.e., establishing external validity and evaluating impact to a broader targeted population and setting; Gitlin & Czaja, 2015).

We recorded the delivery characteristics and adaptation (if any) and tabulated with reference to the body of literature (Objectives B and C). Notably, the same program may be evaluated in different populations and settings across studies; thus, we synthesized the delivery characteristics (i.e., program content, dosage and duration, modality, and setting) across studies when applicable. Adaptation refers to a modification of an efficacious program to meet the needs of its new target population and community context while maintaining fidelity to its core components (Escoffery et al., 2018). We extracted relevant information (i.e., characteristics of populations and settings targeted for program adaptation, reasons and types of modifications, adaptation steps, and evaluation outcomes) to better understand how adaptations of evidence-based intervention have occurred in this literature.

We evaluated the primary and secondary outcomes of each prevention program based on the developmental stages of research represented in each study (Objective D). This enabled a judicious interpretation in the effectiveness of prevention programs for grandparent caregivers. For statistical analysis, we evaluated the efficacy or effectiveness of the programming using Cohen’s *d* effect sizes based on intention-to-treat analysis (Borenstein et al., 2009). We used the effect sizes provided or calculated the effect sizes (if necessary) for primary and secondary outcomes for each programming. We did not synthesize the pooled effect size of each outcome in view of the relatively early stage of intervention development of programming, as well as small sample sizes, and considerable heterogeneity between included studies. However, we categorized the effect sizes based on the identified core components when reporting.

We applied a distillation approach to identify the core components across grandparent programs at or beyond *Phase 2: Pilot testing* (Objective E). We excluded core components under *Phase 1: Feasibility *as the program content and components were still under refinement. The distillation approach identifies the core components and specific elements (i.e., techniques or skills) of the program based on the program manuals (Chorpita et al., 2005). This approach enables the understanding of similarities and differences among programs, and thus to shed lights for gaps and possibilities for new programs based on the current literature. Core components refer to individual treatment practices that comprise a packaged intervention. Because different studies provide different levels of details regarding program and/or session content, we summarized core components (e.g., parenting practices) and labeled specific elements of core components (e.g., problem solving, communication skills) if specified. The first author extracted data from the studies and conducted initial coding. The second author audited the process and checked a subset of studies for agreement in coding. Both authors distilled the specific elements and core components. While there were few disagreements regarding the classification of study variables, any discrepancies in coding were resolved through discussion. In addition, we provided the definition of each core component and specific elements with the corresponding programs to facilitate the conceptualization and future development of core components and specific elements in prevention programs for grandparent caregivers.

## Results

Thirty-five peer-reviewed articles, consisting of 21 programs, were included in this review. The quantity of studies examining grandparent programs has increased over time, with 26 of the 35 articles published in the last 10 years. We reported program characteristics based on a synthesis of multiples studies of the same program (see Table 1). For study characteristics, see Supplementary Table S2.

**Table 1:** Intervention characteristics

Program brand name	Study	Country (region)	Caregiver types	Objectives	Developmental phase of program	Delivery content	Program delivery/ setting	Session/period interval
P1 NA (Small-group school-based intervention)	(Burnette, 1998)	USA (NY)	Custodial caregiver	To access information on effective parenting strategies and stress management	Feasibility	Support group	Group/school	8 sessions/8 weeks/weekly
P2 Project Healthy Grandparents (PHG)	(Kelley et al., 2001, 2007, 2010, 2013, 2019; Kicklighter et al., 2007)	USA (GA)	Custodial caregiver	(a) To reduce psychological stress and improve physical and mental health; (b) to strengthen social support and family resources	Feasibility	Home visit, case management, support group, physical activity, and skill training	Individual/home and group/community	24 sessions/1 year/twice a month
P3 NA (Support group for children with developmental disabilities & delay)	(McCallion et al., 2004)	USA (NY)	Primary/custodial caregiver	To address the unique issues of GPs caring for GC with a developmental disability or delay	Pilot	Case management and Support group	Group/community	6 sessions/12–24 weeks (3–6 months)
P4 NA (Health education intervention with elders having children with HIV/AIDS)	(Boon et al., 2009)	South Africa (Eastern Cape)	Primary caregiver	(a) To enhance ability to cope with their psychosocial needs; (b) to facilitate intergenerational communication between GPs and their dependents; (c) to facilitate the dissemination of information on existing community and social support services	Feasibility	Psychoeducation	Individual/home and group/community	4 sessions/1 month/weekly
P5 Project Grandfamilies Health Watcher	(Hrostowski & Forster, 2010)	USA (MS)	Custodial caregiver	To improve nutrition, physical health and mental health of custodial GPs	Feasibility	Home visit and psychoeducation	Individual/home and group/community	52 sessions/1 year/weekly
P6 Demonstration Project	(Bigbee et al., 2011)	USA (ID)	Custodial & supplementary caregiver	To promote health of caregiving GPs at high-risk families	Feasibility	Home visit	Individual/home/community	6 sessions/6 months/monthly
P7 Grandparenting Case Management (GCM) Program	(Campbell et al., 2012)	USA (CA)	Custodial caregiver	(a) To empower custodial GPs to develop their own abilities; (b) to identify and use resources improving health and well-being of GPs and GC	Feasibility	Case management and skill training	Individual/home and group/community	12 visits, 52 groups/1 year/monthly visit, weekly group
P8 Grandparent Triple P (GTP)	(Kirby & Sander, 2014b; Leung et al., 2014)	Australia & China (Hong Kong)	Supplementary caregiver	To prevent and reduce emotional and behavioral problems in children through promoting parental skills, knowledge, and confidence	Pilot	Skill training	Group/community	6sessions/2 months/weekly
P9 Behavioral parent training (BPT)	(Smith et al., 2016, 2018)	USA (CA, OH, MD, TX)	Custodial caregiver	(a) To improve parenting and reduce stressful child behavior problems; (b) to reduce caregiver distress	Efficacy	Skill training	Group/community	10 sessions/2.5 months/weekly
P10 Cognitive-behavioral therapy (CBT)	(Smith et al., 2016, 2018, 2022)	USA (CA, OH, MD, TX)	Custodial caregiver	(a) To reduce caregiver distress; (b) to improve performance on care-related tasks to which anxiety about failure is attached	Efficacy	Skill training	Group/community	10 sessions/2.5 months/weekly
P11 Biofeedback Control Training	(Zauszniewski & Musil, 2014; Zauszniewski et al., 2013)	USA (OH)	Any caregiver	To reduce stress and depression symptoms by visualizing own bodily symptoms	Feasibility; pilot	Skill training/psychoeducation	Group/community	1 session/1 month follow-up
P12 Resourcefulness Training (RT)	(Musil et al., 2015; Zauszniewski & Musil, 2014; Zauszniewski et al., 2012, 2014a, 2014b, 2017)	USA (OH)	Any caregiver	To help caregivers manage their complex family situations that may have immediate and long-term consequences for themselves and their families	Feasibility; pilot	Skill training/psychoeducation	Online or group/community	1 month follow-up
P13 Child Directed Interaction Training (CDIT)/Parent–Child Interaction Therapy (PCIT)	(N’zi et al., 2016)	USA (FL)	Kinship foster caregiver	(a) To enhance caregiver-child attachment relationship by providing caregivers with concrete skills to increase emotional reciprocity; (b) to reduce foster care child behavior problems	Feasibility	Skill training	Group/community	8 sessions/1 month/twice a week
P14 Intergenerational Physical Activity Intervention	(Young & Sharpe, 2016)	USA (FL)	Supplementary caregiver	(a) To address muscle toning, balance, flexibility, cardio exercise, and mind and body coordination in GP-GC dyads via Zumba dance; (b) to incorporate social components for participant interaction	Feasibility	Physical activity	Group/community	16 sessions/8 weeks/twice a week
P15 GRANDCares Project (Adapted from Powerful tools for Caregivers)	(Yancura et al., 2017)	USA (HI)	Custodial caregiver	To empower Native Hawaiian custodial grandparents to better manage caregiving responsibilities, reduce burnout, and access community resources	Feasibility	Skill training/psychoeducation	Group/community	6 sessions/6 weeks
P16 Trauma-Informed Parenting Class – Resource Parent Curriculum (RPC)	(Foli et al., 2018)	USA (IN)	Primary/custodial caregiver	(a) To build on the strengths and acknowledge the challenges of kinship parenting; (b) to recognize the trauma GC have experienced and provide caregivers with information for traumatized GC	Feasibility	Skill training/Psychoeducation	Group/community	2 sessions/2 weeks/weekly
P17 Conditional Cash Transfer (CCT) Program	(Zhang et al., 2018)	China (Hunan)	Primary caregiver	To enhance nutrition knowledge and food practice behavior of caregivers of left behind children	Efficacy	Psychoeducation	Group/ community	6 sessions/1 year/bi-month
P18 Goal-Setting and Communications Skills Program	(Hayslip et al., 2021; Montoro-Rodriguez & Hayslip, 2019; Montoro-Rodriguez et al., 2021)	USA (TX)	Custodial caregiver	To improve the health and social psychological outcomes for custodial GPs	Feasibility; Pilot	Skill training	Individual/home/community	4 or 6 sessions/4 or 6 weeks/weekly
P19 Active and Healthy Grandchildren	(Xie et al., 2019)	USA (CA)	Supplementary caregiver	To enhance GPs’ knowledge and self-efficacy in supporting GC’s physical activity	Feasibility	Psychoeducation and physical activity	Group/community	4 sessions/ month/weekly
P20 Psychoeducational intervention about autism spectrum disorder (ASD)	(Zakirova‑Engstrand et al., 2021)	Sweden (Stockholm)	Non-custodial caregiver for GC with ASD	(a) To increase GPs’ knowledge of ASD, difficulties and strengths associated with the disorder; (b) to provide information on how GPs can support their GC’s positive development; (c) to discuss GPs’ role in supporting their adult children; and (d) to ensure peer support	Feasibility	Psychoeducation	Group/outpatient clinical setting	1 session
P21 Powerful Tools for Caregivers – Grandfamilies (Adapted from Powerful tools for Caregivers) (PTC-G)	(Fox et al., 2022)	USA (CO, HI)	Primary caregiver	(a) To enhance self-efficacy in grandparents raising grandchildren; (b) To develop self-care tools to reduce stress, recognize emotions, set goals, change negative self-talk, and communicate needs	Feasibility	Skill training/psychoeducation	Group/community	6 sessions/6 weeks/weekly

*GP* = grandparent, *GC* = grandchildren

### Study Characteristics

#### Study Design

Collectively, almost one-half of programs (*n* = 11) adopted pretest–posttest designs with all grandparent caregivers receiving prevention programs (see Table 1). Eight programs adopted RCT designs (i.e., 5 pilot and 3 full-scale efficacy trials) with control conditions that include a waitlist control (*n* = 4), treatment-as-usual (*n* = 2), or an information only condition (*n* = 2). Two programs adopted quasi-experimental designs with a treatment-as-usual condition.

#### Samples

A majority of programs (*n* = 17) were evaluated in the USA (see Table 1), with five programs predominantly targeting Black grandparent caregivers (Burnette, 1998; Campbell et al., 2012; Kelley et al., 2001, 2007, 2010, 2013, 2019; Kicklighter et al., 2007; McCallion et al., 2004; Young & Sharpe, 2016) and single programs targeting Latino caregivers (Xie et al., 2019) and Native Hawaiian caregivers (Yancura et al., 2017), respectively. The remaining four programs were conducted in Australia (Kirby & Sanders, 2014b), China (Xie et al., 2019), Hong Kong, China (Leung et al., 2014), South Africa (Boon et al., 2009), and Sweden (Zakirova‑Engstrand et al., 2021), respectively.

Most programs targeted grandparents who served as primary or custodial caregivers (*n* = 15; see Table 1), while three programs recruited all types of grandparent caregivers. Only three programs specifically targeted supplementary caregivers (Kirby & Sanders, 2014b; Leung et al., 2014; Xie et al., 2019; Zakirova‑Engstrand et al., 2021). Most programs used convenience and/or snowball sampling to recruit primarily grandmother or great-grandmother caregivers caring for grandchildren of any age. Very few grandfather caregivers were recruited in prevention programs. In addition, a majority of grandparent caregivers across programs were of low education (i.e., some high school) and from lower middle-income families.

Furthermore, four programs targeted grandparents of grandchildren with special needs and/or health conditions, including orphans having HIV/AIDS (Boon et al., 2009), preschool-aged children with autism spectrum disorder (Zakirova‑Engstrand et al., 2021), youth with developmental disabilities and delays (McCallion et al., 2004), and youth with behavior problems and a history of maltreatment (N’zi et al., 2016).

### Program Characteristics

#### Developmental Stages of Studies Evaluating Programs

Most grandparent programs were evaluated in early developmental stages of research (Table 1**)**. Twelve programs had not progressed beyond feasibility studies. Researchers evaluated these programs for their feasibility and acceptability of the program content and delivery modality. The program content focused on improving caregiving knowledge, perceptions of caregiving, and different health outcomes of grandparent caregivers. Notably, most feasibility studies recruited small sample sizes, with fewer than 25 caregiver participants.

Eight grandparent programs were evaluated using RCT designs. Among those, five grandparent programs had gone through pilot RCTs to examine the feasibility and potential treatment effects using control groups. Two programs were evaluated in multiple RCTs. For example, Resourcefulness Training was examined in different delivery modalities (i.e., in person and online; Musil et al., 2015; Zauszniewski et al., 2014a) and outcome assessment methods (i.e., reflective journaling and voice recording; Zauszniewski et al., 2014a). Moreover, three programs were advanced to efficacy studies to examine outcomes relative to a comparison or control group. For example, Smith et al. (2016, 2022) examined the patterns of enrollment and engagement of custodial grandparents in a three-arm RCT and differential efficacy of two interventions on mental health according to caregiver characteristics.

#### Modes of Program Delivery

Grandparent programs were administered using different modalities (e.g., individual-based, group-based, website administration) in different settings (e.g., home, school, and community). Most programs included weekly sessions, varying in total duration from 1 week up to 1 year. The number of sessions ranged from one to 64 (*M* = 12.9, *SD* = 17.4). Moreover, most programs (*n* = 11) were administered in community settings only. Relatively few programs were delivered in home (*n* = 4) or school (*n* = 1) setting. More than half of the programs were delivered in a group format (*n* = 9), while two programs were delivered in an individual format. Four programs were delivered in a mix of individual and group formats. Finally, Resourcefulness Training was adapted using a web-based administration (Musil et al., 2015).

### Adaptations

Five grandparent programs included adaptations that were evaluated for feasibility with grandparent caregivers. Of those five programs, four programs reported adaptations to a new target population in the USA, while one program reported adaptations for populations in two different regions, Australia and Hong Kong, China. The main reasons for adaptation included focusing on a new target population of family caregivers (*n* = 4) and the need for cultural appropriateness (*n* = 1). The common adaptations were content modification due to new target population (*n* = 3), cultural modifications (*n* = 1), and both content and cultural adaptation (*n* = 1). For content modification, one program was modified for grandparents from the original focus on parent caregivers (Kirby & Sanders, 2014b; N’zi et al., 2016), and another from the original focus on family caregivers for adults with chronic diseases (Fox et al., 2022). For cultural adaptation, two groups of researchers adapted grandparent programs to be used in a different country (i.e., Hong Kong, China; originally developed in Australia; Leung et al., 2014) and for new ethnic/racial groups (i.e., Native Hawaiian; originally developed for non-Hispanic White American; Yancura et al., 2017).

### Program Outcomes by Stages of Development

#### Feasibility Studies

Thirteen newly created or adapted grandparent programs were evaluated for feasibility in one or more domains (e.g., ability to recruit participants, delivery of all session content with fidelity, acceptability of delivery format, acceptable completion of the program by participants) using both quantitative and qualitative methods. Nine programs were examined for the feasibility of their delivery formats (i.e., group, home visit, case management, and multi-component) for grandparent caregivers. Three programs examined the feasibility of adapting existing evidence-based interventions designed for family caregivers to a new target population of grandparent caregivers (Fox et al., 2022; N’zi et al., 2016; Yancura et al., 2017). One study examined the acceptability of trauma-informed approach among grandparent caregivers (Foli et al., 2018). Another study examined the feasibility of online delivery (Musil et al., 2015).

#### Pilot Randomized Controlled Trials

Although studies employing pilot RCTs examined preliminary outcomes, effect size estimates from small sample size should be treated cautiously due to the unreliability of the estimates (Kistin & Silverstein, 2015). Additional studies are needed in order to more reliably examine program efficacy and generalizability. In the context of these pilot RCTs, four different grandparent programs demonstrated medium to large effect sizes in three areas, including increasing parenting efficacy, reducing psychological distress and increasing the use of proactive coping strategies (Kirby & Sanders, 2014b; Leung et al., 2014; McCallion et al., 2004; Montoro-Rodriguez et al., 2021; Zauszniewski et al., 2013, 2014a). In addition, one grandparent program, respectively, demonstrated a large effect size in boosting quality of life (Zauszniewski & Musil, 2014) and a medium effect size in reducing grandchildren’s disruptive behavior (Kirby & Sanders, 2014b).

#### Full-Scale Efficacy Trials in Community Settings

Two full-scale efficacy trials in community settings demonstrated statistically significant effects in three domains of outcomes. Compared to pilot RCTs, the evaluation of effect sizes in efficacy trials is relatively reliable given adequate sample sizes and high internal validity. Grandparent programs demonstrated a medium effect size in reducing coercive parenting (Smith et al., 2018), a medium effect size in improving parenting knowledge (Zhang et al., 2018), and a medium effect size in improving mental health symptoms (Smith et al., 2018). Two programs demonstrated a large effect size in reducing grandchildren behavioral problems (Smith et al., 2018). Moreover, older age and higher attendance of treatment sessions of grandparent caregivers was associated with treatment satisfaction and effectiveness in reducing caregiver distress (Smith et al., 2016, 2022).

#### Content and Core Components of Programs

Five programs were comprehensive multi-component programs consisting of psychoeducation, skill training, and case management (Campbell et al., 2012; Hrostowski & Forster, 2010; Kelley et al., 2001; McCallion et al., 2004; Xie et al., 2019). A majority of programs (*n* = 9) were psychoeducational or utilized skill-based training, while one program was solely a support group (Burnette, 1998). Notably, five programs offering personalized content targeted custodial caregivers (Campbell et al., 2012; Hrostowski & Forster, 2010; Kelley et al., 2001, 2007, 2010, 2013; McCallion et al., 2004) or grandparent caregivers with grandchildren experiencing developmental problems (Bigbee et al., 2011).

We identified six core components and 17 specific elements among eight grandparent programs at or beyond pilot RCTs (see Table 2). The six core components included *parenting knowledge and skills*, *adaptive coping strategies*, *addressing physical health needs of grandparents*, *mobilizing and expanding personal social support*, *accessing social services*, and *managing interpersonal family relationships and conflicts*.

**Table 2:** Core components and specific elements of randomized-control trial prevention programs for grandparent caregivers

Core component^a^	Specific element^a^	Definition	Program reference^b^
**Parenting knowledge and skills**	-	Learning general parenting knowledge and skills interacting with a new generation of children	P3, P8, P9, P10, P12, P18, P19
	Child development knowledge	Learning knowledge of child and youth development	P8, P18
	Managing GC with special needs	Learning knowledge and skills in caring GC of special education, social and emotional needs	P3
	Setting childrearing goals	Identifying short-term goals to achieve (e.g., personal, social, and relationship-oriented aspects of caregiving)	P8, P19
	Positive grandparenting	Learning to be a positive GP by attending to desirable GC behavior and reinforcing with reward/praise	P8, P9, P10
	Relationship building	Learning how to build up positive relationship with GC through emotional reciprocity	P8
	Problem-solving skills	Generating and implementing solutions to difficult parenting situations	P8, P10, P12
	Communication skills	Learning attitude toward and effective communication skills with GC	P8, P9, P19
	Rule setting	Setting clear rules about appropriate and inappropriate behavior	P8, P9
	Non-violent disciplining	Reacting to disruptive GC behavior with a nonviolent consequence aiming to reduce the behavior	P3, P8, P10
	Proactive contingency planning for GC’s future	Discussing concerns about GC in the future and setting contingency plan of childcare	P3, P8, P19
**Adaptive coping strategies**	-	Learning coping strategies to manage stress and unhelpful emotions	P3, P8, P10, P11, P12
	Self-care	Building skills of self-care (e.g., respite)	P3
	Identifying common stressors and their impact	Recognizing own feelings and emotions as caregivers, and the impact on their family relationships	P8, P10
	Positive self-talk		P12
	Relaxation technique	Demonstration and practice of relaxation technique	P3, P10
	Using biofeedback equipment	Using biofeedback equipment to visualize own physiological reactions to stress and negative emotions	P11
	Cognitive reframing	Identifying and hanging the perception of parenting experiences	P10, P12
	Reflective journaling	Completing a journal about daily events, thoughts and feelings about their day with GC	P11, P12
**Addressing physical health needs of GPs**	Psychoeducation on GP’s physical health needs	Health promotion about nutrition, exercise, sleep, and smoking cessation	P3
**Mobilizing and expanding personal social support**	-	Mapping social support network, including family, friends, and community, in providing care to GC	P12, P19
**Accessing social services**	-	Providing assistance for social services and resources, such as legal assistance and entitlement for GC care, referral to child and social services, etc	P3
**Managing intergenerational family relationships and conflicts**	-	Learning how to manage emotional distress from dysfunctional parents (i.e., adult children), role conflicts of GPs, collaborating with parents to promote healthy GC development, and divided loyalties of GC	P8

*GP* = grandparent, *GC* = grandchildren

^a^Core components are provided for grandparent programs at or beyond pilot randomized-controlled trials, while specific elements are provided if listed in the included articles

^b^Program reference: P3, N.A. (support group for children with developmental disabilities and delay); P8, grandparent Triple P (GTP); P9, behavioral parent training (BPT); P10, cognitive-behavioral therapy (CBT); P11, biofeedback control training; P12, resourcefulness training; P18, conditional cash transfer (cct) program; P19, goal-setting and communications skills program

*The core component of parenting knowledge and skills* was identified in eight programs (100%). Some common specific elements of this component were *positive grandparenting*, *problem-solving skills*, *communication skills*, use of *non-violent disciplining,* and *proactive contingency planning for the future of grandchildren*. Five programs (62.5%) included the core component of *adaptive coping strategies*, which taught grandparent caregivers coping strategies to manage stress and unhelpful emotions. The component included specific elements such as *identifying common stressors and their impact*, *relaxation techniques*, *cognitive reframing*, and *reflective journaling*. Another core component was *addressing the physical health needs of grandparents* (*n* = 1). Specifically, the program provided health-related psychoeducation, such as nutrition, exercise, sleep, and smoking cessation.

Moreover, two programs (25%) which targeted custodial or primary caregivers offered the core component of *mobilizing and expending personal social support* in family and friends, and one program provided assistance for *accessing social services* such as legal assistance and entitlement programs for grandchildren. In navigating relationships between adult children and grandchildren, a unique core component which appeared in one program targeting supplementary caregivers was *managing intergenerational family relationships and conflicts*. This core component aimed to better align grandparents and parents as a family team to promote the development of grandchildren collaboratively with a goal of increasing the consistency of parenting and reducing parenting-related conflict across caregivers.

## Discussion

This systematic review provides an in-depth evaluation of the wide range of prevention programs for grandparent caregivers according to the developmental stages of research framework (Gitlin & Czaja, 2015). All grandparent programs were preventative in nature and designed to promote positive grandparenting and the well-being of grandparent caregivers as agents of change for grandchild development. This systematic review examined not only evidence for feasibility and efficacy of grandparent programming, but also identified the core components, delivery methods, and evidence of adaptation.

We identified a number of limitations in the current state of grandparent programming. First, a majority of studies targeted grandparents who served as primary or custodial caregivers, but not supplementary caregivers. Second, the present studies lack international coverage, primarily conducted in the USA. Third, over half of the programs had not progressed beyond early stages of development as feasibility studies and without controlled comparison groups. A limited number of grandparent programs were evaluated in efficacy or effectiveness trials. Last, almost all grandparent programs evaluated immediate outcomes of grandparent caregivers and grandchildren (i.e., one-month up to one-year after the intervention). These limited our understanding of the stability of grandparent programs and the crossover effect from grandparent caregivers to grandchildren.

Despite the limitations in existing study designs, this review informs and advances the development and evaluation of grandparent programs. The evidence of program adaptation suggests that programming may be successfully adapted to meet the different needs of grandparent caregivers globally. Feasibility and acceptability were demonstrated in most pretest–posttest design feasibility and pilot RCT studies, while full-scale efficacy trial studies demonstrated promising outcomes in reducing negative parenting and caregiving distress among grandparent caregivers. This body of literature includes a number of programs with promising feasibility that may be next evaluated in rigorous RCTs as well programs with strong evidence of efficacy that may be next evaluated for effectiveness under real-world practice conditions. We identified six common core components across eight grandparent programs evaluated through pilot RCT or efficacy testing.

### Program Delivery and Diversity Among Caregivers

It is important for future researchers to evaluate dosage effects on program outcomes. Given the wide range of dosage across grandparent programs, it appears that at-risk custodial caregivers may benefit from medium-length programs with intense follow-ups, while typical supplementary grandparent caregivers may need briefer programs with sparse enhancement sessions. Future research should explore the optimal intervention dose before large-scale dissemination and implementation.

Importantly, the delivery format is another key consideration to enhance the attendance and engagement of grandparent caregivers. One skill-based training was administered by a website, targeting well-educated primary grandparent caregivers (Musil et al., 2015). Web-based delivery methods may be an important future trend, particularly considering social distancing trends launched during the COVID-19 pandemic. Website administration also offers the benefit of accessibility to caregivers. Future research may examine the groups or subgroups of grandparent caregivers who are most likely to enroll and engage in website or mobile app-based programming. Nevertheless, future researchers should pay attention to the availability of digital devices and computer literacy for grandparent caregivers. Potentially, collaborative intergenerational digital learning may be added to programming to promote grandparent-grandchildren relationships.

### Program Adaptation

Emerging grandparent programs have been adapted from existing evidence-based programs in consideration of the unique needs of grandparent caregivers, family structures, and cultural contexts. Adaptations were performed to tailor programming for new target populations of grandparent caregivers (from the original parent or family caregivers) and/or grandparent caregivers in different countries and/or racial/ethnic groups. For example, when adapting parent-focused program for custodial caregivers, Kicklighter et al. (2009) emphasized grandparent caregivers as agents of change for grandchild development by promoting grandparents’ physical well-being (i.e., self-care). When adapting grandparent programs for supplementary caregivers, grandparent caregivers were considered as the agents of change in collaborating with parent caregivers as a family team to support grandchild development (Fox et al., 2022; Kirby & Sanders, 2014b). Moreover, different researcher groups performed cultural adaptations to enhance program engagement and minimize any adverse effects for families of color in the USA (Kicklighter et al., 2009; Xie et al., 2019; Yancura et al., 2017) and outside the program’s initial country of development (Leung et al., 2014).

Future researchers should expand the scope of grandparent programs for supplementary caregivers. As dual-earner families become increasingly common globally, many parents would rather have their parents (i.e., grandparents) care for their children rather than institutional or nonrelative care (Shwalb & Hossain, 2017). Thus, it is important to expand family-focused programs to support the coordination and co-parenting of parents and grandparent caregivers as agents of change for grandchild development. Future researchers should also consider targeting multigenerational families with both parent and grandparent components, such as addressing the relationship between parents and grandparents in the family system. The family systems approach is particularly salient in cultures where grandparents are highly respected as family authority figures and tend to be active in supporting the parenting of parent caregivers (Hoang et al., 2022). The support of multigenerational communication and coordination targeting both grandparent and parent caregivers as agents of change could potentially minimize multigenerational conflicts. Greater consistency in parenting among caregivers will also promote grandchildren’s positive adjustment (Hoang et al., 2022).

Given the limited programming currently available, it will be important to expand grandparent programs for caregivers of color in the USA and community contexts outside the USA. More cultural adaptation of programs based on linguistic, developmental, cultural, and contextual differences of grandfamilies is needed (i.e., one size does not fit all). Grandparents of color (e.g., Native American, Black, and Hispanic) are disproportionately more likely to be raising grandchildren in the grandparents’ middle adulthood (Hadfield, 2014); however, relatively few programs have been adapted to the unique needs and cultural values of these populations. While we have an emerging understanding of grandparenting and its impact on the well-being of caregivers in Asia Pacific and Europe (i.e., regions embracing collectivistic cultures; Chan et al., 2023), there is an empirical gap in adapting grandparent programs outside the USA. In general, successful adaptation of grandparent programs involves striking the right balance between fit and fidelity by making thoughtful, appropriate modification for the needs of grandparent caregivers while preserving the theoretical and practical essence of the intervention.

### Emerging Program Development and Program Efficacy

Most studies and grandparent programs were in the early stages of development. Over half of the programs were evaluated only in pretest–posttest designs, generally demonstrating high feasibility and acceptability by grandparent caregivers. To move forward, programs determined to have high acceptability and feasibility in feasibility studies should be evaluated in vigorous RCTs to determine the causal effects of programming on targeted outcomes. In addition, quantitative findings from pilot RCTs need to be interpreted with caution due to unreliable effect sizes. Only large program effects may emerge as significant due to limited power. Nevertheless, four programs evaluated in pilot RCTs demonstrated preliminary efficacy in the identified domains targeted by core components of the programming. It will be important for researchers to utilize fully powered RCTs to evaluate program efficacy in future work. Furthermore, programs will benefit from evaluation under effectiveness trials in diverse populations and real-world conditions before large-scale implementation and dissemination research. This field will also benefit from collaboration between research laboratories and community-based institutions to evaluate existing community-based programs.

Importantly, future research will benefit from more closely examining the conceptualized mechanisms of grandparent programs (i.e., positing grandparent caregivers as agents of change; Fig. 1) and family-focused programs (i.e., positing grandparent and parent caregivers as agents of change) using rigorous methods. To our best knowledge, no empirical intervention studies have examined whether changes in grandparent caregivers produce subsequent changes in the adjustment of grandchildren. Therefore, it is important to examine the long-term effects of grandparent programs (e.g., beyond 6 months after program) on outcomes of both grandparent caregivers and grandchildren. Grandchild behavior may take additional time to change following changes by grandparent caregivers (Fosco et al., 2021). Moreover, refresher or booster grandparent programs may be required in anticipation of some declines in parenting skills that may be evident particularly in high-risk custodial caregivers (Fosco et al., 2021). Second, most outcomes were self-reported by grandparent caregivers; future research will benefit from a multi-method and multi-informant approach, such as clinicians’ report of health or physiological indicators of grandparent caregivers’ health, or family observations of grandparent-grandchild interactions.

### Core Components of RCT Programs

Six core components were identified in grandparent programs. While four identified core components are similar to parent-focused or family caregiver-focused programs, *addressing physical health needs* and *managing intergenerational family relationships and conflicts* were distinct for grandparent caregivers.

*Parenting knowledge and skills* and *adaptive coping strategies* were two major core components identified in most grandparent programs. Two distinct parenting elements specific to grandparent caregivers were *communication skills with grandchildren* and *developing proactive contingency plans for grandchildren’s future* (Leijten et al., 2019). Similar to prevention programs for families with multiple problems (Visscher et al., 2018), programs for grandparent caregivers included core components of *mobilizing and expanding personal social support* and *accessing social services*. These core components were relevant for custodial caregivers, whose adult children or grandchildren might be involved in social services systems (e.g., financial or legal assistance).

Given the wide age range of grandparent caregivers (mid to late adulthood), *addressing physical health needs* was also a unique core component in grandparent programming. As grandparent caregivers are viewed as agents of change, the maintaining of health ensure family stability and thus enhance the well-being of grandchildren in the long-term. Importantly, *managing intergenerational family relationships and conflicts* represented another distinct core component found in programs for supplementary caregivers that was not typically included in traditional programs for parent caregivers (Kirby & Sander, 2014b; Leung et al., 2014).

This review is the first to identify the core components of grandparent programs. Because multicomponent programming may contain components that vary in their individual effectiveness, future researchers are encouraged to conduct randomized controlled micro-trials to identify efficacious core components in multi-component programming. This research design enables researchers to evaluate hypotheses about the efficacy of specific skills or activities for grandparent caregivers as well as examine potential moderators of effects (i.e., how efficacy may vary for caregivers with different characteristics). For example, we may examine whether it is efficacious to include *addressing physical health needs* for grandparent caregivers of different age and care intensity. By identifying active ingredients for specific types of grandparent caregivers (i.e., what works best for whom), the inactive ingredients can be eliminated. This helps shorten the length of programs while reducing costs and supporting dissemination on a larger scale.

## Conclusion

This systematic review seeks to better solidify our current understanding of the state of grandparent programming, with a goal of informing and advancing the development and evaluation of programming in this area. The emerging evidence broadly supports the use of prevention programming supporting the distinct needs of grandparent caregivers raising grandchildren. We have conceptualized a potential mechanism of existing grandparent programs—targeting grandparent caregivers as the agents of change for the development of grandchildren. However, most existing grandparent programs are in the early stages of development. To move this body of research forward, future researchers should conduct vigorous RCTs with adequate power, examine the long-term effects of programs, and adopt measures using a multi-method and multi-informant approach. Further analyses should be conducted to examine the mechanisms of existing grandparent programs using longitudinal mediation methods. To streamline grandparent programs, future researchers should examine which core components of programs work for which types of grandparent caregivers and under which types of delivery strategies. This will effectively and efficiently promote positive grandparenting and the well-being of grandfamilies of diverse needs.

### Supplementary Information

Below is the link to the electronic supplementary material.

Supplementary file1 (DOCX 86 KB)
