# Supplementary material for: Evaluation of Prevention Programs for Grandparent Caregivers: a Systematic Review
Source: Prev Sci. 2023 Oct 14;25(1):137–54. doi: 10.1007/s11121-023-01594-2 (PMC10830810; doi:10.1007/s11121-023-01594-2)
Supplement: Supplementary file 1 — Supplementary file1 (DOCX 86 KB) [file 11121_2023_1594_MOESM1_ESM.docx]

**Supplemental Materials**

**Figure S1** *Flow Diagram of Intervention for Grandparent Caregivers Literature Search and Inclusion Process.*

## **Screening**

## **Inclusion**

## **Eligibility**

## **Identification**

Records identified through database searching
(*n* = 1970)

Additional records identified through other sources
(*n* = 6)

Records after duplicates removed
(*n* = 1483)

Abstract screening
(*n* = 1483)

)

Records excluded (*n* =1407):

- Non peer-reviewed study (*n* = 101)
- Non-empirical study (*n* = 136 )
- Non-intervention study (*n* = 1072)
- Intervention not involving grandparent caregivers (*n* = 98)

Full-text review
for eligibility
(*n* = 76)

Full-text articles excluded (*n* = 41):

- Non RCT/quasi-experiment/pre-post (*n* = 25)
- Intervention targeting kinship caregivers (*n* = 13)
- Intervention not targeting grandparents (*n* = 2)
- Not evaluating relevant outcomes (*n* =11)

Records included
(*n* = 35 articles, describing 21 interventions)

))

**Table S1**. Study Quality Assessment.

| Studies / Criteria^a^ | 1 | 2 | 3 | 4 | 5 | 6 | 7 | 8 | 9 | 10 | 11 | 12 |  |  | Yes | Percent |
| --- | --- | --- | --- | --- | --- | --- | --- | --- | --- | --- | --- | --- | --- | --- | --- | --- |
| **Pretest-posttest** **studies with no control group (*n* = 21)** | | | | | | | | | | | | | | | | |
| S1 Burnette (1998) | Y | N | Y | NR | N | Y | Y | NA | NR | Y | N | NA |  |  | 5 | 50% |
| S2 Kelley et al. (2001) | Y | Y | Y | Y | N | Y | Y | NA | NR | Y | N | NA |  |  | 7 | 70% |
| S3 Kicklighter et al. (2007) | Y | Y | Y | Y | N | Y | Y | NA | NR | Y | N | NA |  |  | 7 | 70% |
| S4 Kelley et al. (2007) | Y | Y | Y | Y | Y | Y | Y | NA | NR | Y | N | NA |  |  | 8 | 80% |
| S5 Kelley et al. (2010) | Y | Y | Y | Y | Y | Y | Y | NA | NR | Y | N | NA |  |  | 8 | 80% |
| S6 Kelley et al. (2013) | Y | Y | Y | Y | Y | Y | Y | N | NR | Y | N | NA |  |  | 8 | 73% |
| S7 Kelley et al. (2019) | Y | Y | Y | Y | Y | Y | Y | NA | N | Y | N | NA |  |  | 8 | 80% |
| S9 Boon et al. (2009) | Y | Y | Y | Y | Y | Y | Y | NA | NR | Y | Y | NA |  |  | 9 | 90% |
| S10 Hrostowski and Forster (2010) | Y | Y | Y | Y | N | Y | Y | NA | NR | N | N | NA |  |  | 6 | 60% |
| S11 Bigbee et al. (2011) | Y | Y | Y | Y | N | Y | Y | N | NR | Y | Y | NA |  |  | 8 | 73% |
| S12 Campbell et al. (2012) | Y | Y | Y | Y | Y | Y | Y | NA | NR | Y | N | NA |  |  | 8 | 80% |
| S19 Zauszniewski et al. (2013) | Y | Y | Y | Y | N | Y | Y | NA | NR | Y | Y | NA |  |  | 8 | 80% |
| S23 Musil et al. (2015) | Y | Y | Y | Y | N | Y | Y | NA | NR | Y | N | NA |  |  | 7 | 70% |
| S24 Zauszniewski et al. (2017) | Y | Y | Y | Y | N | Y | Y | N | Y | NA | N | NA |  |  | 7 | 70% |
| S26 Young & Sharpe (2016) | Y | Y | Y | Y | N | Y | Y | NA | N | NA | N | NA |  |  | 6 | 67% |
| S27 Yancura et al. (2017) | Y | Y | Y | Y | N | Y | Y | N | N | NA | N | NA |  |  | 7 | 70% |
| S28 Foli et al. (2018) | Y | Y | Y | Y | N | Y | Y | NA | NR | Y | N | NA |  |  | 7 | 70% |
| S30 Montoro-Rodriguez and Hayslip (2019) | Y | Y | Y | Y | N | Y | Y | NA | NR | Y | N | NA |  |  | 7 | 70% |
| S33 Xie et al. (2019) | Y | Y | Y | Y | N | Y | Y | NA | Y | Y | N | NA |  |  | 8 | 80% |
| S34 Zakirova‑Engstrand et al. (2021) | Y | Y | Y | Y | Y | Y | Y | NA | Y | Y | Y | NA |  |  | 10 | 100% |
| S35 Fox et al. (2022) | Y | Y | Y | Y | Y | Y | Y | NA | N | Y | Y | NA |  |  | 9 | 90% |
| Studies / Criteria^b^ | 1 | 2 | 3 | 4 | 5 | 6 | 7 | 8 | 9 | 10 | 11 | 12 | 13 | 14 | Yes | Percent |
| **Quasi-experiment (*n* = 4)** | | | | | | | | | | | | | | | | |
| S18 Zauszniewski et al. (2012) | Y | N | NA | NA | NA | NR | Y | N | Y | Y | Y | N | Y | Y | 7 | 64% |
| S20 Zauszniewski et al. (2014a) | Y | NA | NA | NA | NA | NR | NR | NR | Y | Y | Y | N | Y | Y | 6 | 60% |
| S21 Zauszniewski et al. (2014b) | Y | N | NA | NA | NA | Y | NR | NR | Y | Y | Y | N | Y | Y | 7 | 64% |
| S22 Zauszniewski and Musil (2014) | Y | NA | NA | NA | NA | NR | NR | NR | NR | Y | Y | N | Y | Y | 5 | 50% |
| **Randomized-controlled trials (*n* = 10)** | | | | | | | | | | | | | | | | |
| S8 McCallion et al. (2004) | Y | N | N | N | NA | NR | NR | NR | NR | Y | Y | N | Y | Y | 5 | 38% |
| S13 Kirby and Sanders (2014) | Y | N | N | N | NA | Y | Y | Y | NR | Y | Y | N | Y | Y | 8 | 62% |
| S14 Leung et al. (2014) | Y | Y | Y | N | NA | Y | Y | Y | Y | Y | Y | Y | Y | Y | 12 | 92% |
| S15 Smith et al. (2016) | Y | N | N | N | NA | Y | NR | NR | Y | Y | Y | N | Y | Y | 7 | 54% |
| S16 Smith et al. (2018) | Y | Y | Y | N | NA | Y | Y | Y | Y | Y | Y | Y | Y | Y | 12 | 92% |
| S17 Smith et al. (2022) | Y | N | Y | N | NA | NR | NR | NR | Y | Y | Y | Y | Y | Y | 8 | 57% |
| S25 N’zi et al. (2016) | Y | N | N | N | N | Y | Y | Y | Y | Y | Y | N | Y | Y | 9 | 64% |
| S29 Zhang et al. (2018) | Y | Y | Y | N | NA | Y | Y | Y | NR | Y | Y | Y | Y | Y | 11 | 85% |
| S31Hayslip et al. (2021) | Y | N | N | N | NA | NR | NR | NR | NR | NR | Y | Y | Y | Y | 5 | 42% |
| S32 Montoro-Rodriguez et al. (2021) | Y | Y | N | N | NA | Y | N | N | NR | NR | Y | Y | Y | Y | 7 | 58% |

*Notes*. Y = Yes, meeting the criteria, N = No, not meeting the criteria; NR = Not reported; NA = not applicable; Percent = Percentage of Yes out of applicable criteria.

^a^Criteria for Pretest-posttest studies with no control group (See **Appendix 2**): 1 – Clear study question/objective; 2 – Clear selection criteria; 3 – Representative sample; 4 – Participants eligibility; 5 – Adequate effect size; 6 – Clearly described intervention; 7 – Clearly defined outcomes; 8 – Blind to outcome evaluation; 9 – Attrition; 10 – Vigorous pretest/posttest statistical analysis; 11 – Multiple time point measurement; 12 – Clustered data

^b^14-criteria for Pretest-posttest studies with no control group (See **Appendix 2**): 1 – Clear description of a RCT; 2 – Adequate randomization; 3 – Concealed treatment allocation; 4 – Double-blind assignment; 6 – Blind to outcome evaluation; 7 – Attrition; 8 – Differential dropout rate; 9 – Fidelity; 10 – Controlled other treatments; 11 – Vigorous outcome evaluation; 12 – Adequate sample size; 13 – Prespecified subgroup analyses; 14 – Intention-to-treat

**Table S2**. Summary of 35 Included Studies.

| Study | Program brand name | Stage of development | Study design | Country/ Region | Intervention group size | Control group size | Grandparent (GP) age, gender, and ethnicity | GP caregiver type | Grandchild (GC) cared for, age, and special needs |
| --- | --- | --- | --- | --- | --- | --- | --- | --- | --- |
| S1 Burnette (1998) | P1 N.A. (Small-group school-based intervention) | Feasibility | Pretest-posttest | U.S. (NY) | 11 | NA | Age *M* = 56.5 (*SD* = 6.4); 91% female; 91% Black, 9% Latino; 91% at least some high school | Custodial caregiver | 3 GC; Age *M* = 9.0 (*SD* = 3.6); 33% special physical, social, emotional, and/or educational needs |
| S2 Kelley et al. (2001) | P2 Project Healthy Grandparents (PHG) | Feasibility | Pretest-posttest | U.S. (GA) | 24 | NA | Age *M* = 55.7 (*SD* = 10.5); 96% female; 100% Black; 56% some high school, 40% high school graduate, 4% some college | 38% had guardianship, 23% had no legal relationship, 20% had custody, 17% cared in a state custody; 2% adopted GC | 2.7 GC; Age *M* = 6.8 (*SD* = 3.9) |
| S3 Kicklighter et al. (2007) | P2 PHG | Feasibility | Pretest-posttest | U.S. (GA) | 22 | NA | Age *M* = 61 (*SD* = 6.1); 86% female; 100% Black | Primary caregiver | 2.3 GC; Age *M* = 12.3 |
| S4 Kelley et al. (2007) | P2 PHG | Feasibility | Pretest-posttest | U.S. (GA) | 120 | NA | Age *M* = 56.1 (*SD* = 8.9); 100% female; 100% Black; 47% some high school, 28% high school graduate, 26% some college or above | 11% formal kinship care; 89% informal kinship care | 2.5 GC; Age *M* = 8.7 (*SD* = 4.7) |
| S5 Kelley et al. (2010) | P2 PHG | Feasibility | Pretest-posttest | U.S. (GA) | 529 | NA | Age *M* = 56.7 (*SD* = 8.9); 100% female; 99% Black, 1.5% White or Hispanic; 53% less than high school | Custodial caregiver | 2.5 GC; Age *M* = 4.2 (*SD* = 3.4) |
| S6 Kelley et al. (2013) | P2 PHG | Feasibility | Pretest-posttest | U.S. (GA) | 504 | NA | Age *M* = 56.7 (*SD* = 8.9); 100% female; 100% African American; 52% less than high school | Custodial caregiver | \| 2.5 GC; Age  *M* = 4.2 (*SD* =  3.4) \| \| --- \| |
| S7 Kelley et al. (2019) | P2 PHG | Feasibility | Pretest-posttest | U.S. (GA) | 549 | NA | Age *M* = 56.3 (*SD* = 8.8); 100% female; 100% Black; 61% less than high school | Custodial caregiver | \| 2.5 GC; Age  *M* = 8.2 (*SD* =  4.1) \| \| --- \| |
| S8 McCallion et al. (2004) | P3 N.A. (Support group for children with developmental disabilities and delay) | Pilot | RCT | U.S. (NY) | 49 | 48 | Age *M* = 56.7 (*SD* = 8.9); 94% female; 79% Black; 25% some college or more | 75% custodial caregiver; 24% primary caregiver | 1.7 GC; Age *M* = 11; Developmental disabilities and delays |
| S9 Boon et al. (2009) | P4 N.A. (Health education intervention with elders having children with HIV/AIDS) | Feasibility | Pretest-posttest | South Africa (Eastern Cape) | 202 | NA | Age ≥ 60 (39% 60-69, 36% 70-79, 23% ≥ 80); 82% female; 100% isiXhosa; 32% some primary school, 10% some high school, 58% missing | Primary caregiver | Orphans with HIV and AIDS |
| S10 Hrostowski and Forster (2010) | P5 Project Grandfamilies Health Watcher | Feasibility | Pretest-posttest | U.S. (MS) | 18 | NA | Age *M* = 65; 85% female; 56% Black, 44% White; 17% some high school, 39% high school graduate, 44% some college | Custodial caregiver | 2 GC; Age ranged 6-17 |
| S11 Bigbee et al. (2011) | P6 Demonstration Project | Feasibility | Pretest-posttest | U.S. (ID) | 11 | NA | Age *M* = 60.5; 55% female; 100% White; 64% high school graduate | 55% custodial and 45% supplementary caregiver | 2.4 GC; Age *M* = 8.1 (*range* = 9 months to 19 years); Some with significant emotional, developmental, and/or physical health concerns |
| S12 Campbell et al. (2012) | P7 Grandparenting Case Management (GCM) Program | Feasibility | Pretest-posttest | U.S. (CA) | 50 | NA | Age *M* = 56 (*range* = 35-77); 83% Black; 33% some high school | Custodial caregiver | 2.3 GC; School age |
| S13 Kirby and Sanders (2014b) | P8 Grandparent Triple P (GTP) | Pilot | RCT | Australia | 28 | 26 | Age *M* = 60.9; 94% female; 96% White 30% high school or less, 70% some college | Supplementary caregiver | 2.4 GC; Age *M* = 4.4 (*SD* = 2.3) |
| S14 Leung et al. (2014) | P8 GTP | Pilot | RCT | China (Hong Kong) | 29 | 27 | Age *M* = 61.7 (*SD* = 7.1); 80% female; 100% Chinese; 50% elementary school or below; 50% high school or above | Supplementary caregiver | Age *M* = 3.8 (*SD* = 1.1) |
| S15 Smith et al. (2016) | P9 Behavioral parent training (BPT); P10 Cognitive-behavioral therapy (CBT) | Efficacy | RCT | U.S. (CA, OH, MD, TX) | BPT=115, CBT=128 | 100 | Age *M* = 58.4 (*SD* = 8.2); 80% female; 49% White, 43% Black; 15% some high school or below, 21% high school graduate, 65% some college or above | Custodial caregiver | 1.8 GC; Age *M* = 7.8 (*SD* = 2.5) |
| S16 Smith et al. (2018) | P9 BPT; P10 CBT | Efficacy | RCT | U.S. (CA, OH, MD, TX) | BPT=115, CBT=128 | 100 | Age *M* = 58.4 (*SD* = 8.2); 80% female; 49% White, 43% Black; 15% some high school or below, 21% high school graduate, 65% some college or above | Custodial caregiver | 1.8 GC; Age *M* = 7.8 (*SD* = 2.5) |
| S17 Smith et al. (2022) | P9 BPT; P10 CBT | Efficacy | RCT | U.S. (CA, OH, MD, TX) | BPT=115, CBT=128 | 100 | Age *M* = 58.4 (*SD* = 8.2); 80% female; 44% White, 43% Black; 64% some college or above | Custodial caregiver | Age *M* = 7.8 (*SD* = 2.6) |
| S18 Zauszniewski et al. (2012) | P12 Resourcefulness training (RT) | Pilot | Quasi-experiment | U.S. (OH) | 40 (expression with RT) | 41 (expression only)  21 (control) | Age *M* = 58 (*range* = 40-82); 100% female; 9% less than high school, 31% high school/GED, 60% some college or above | Any caregiver | Age ranged 6 months – 18 years |
| S19 Zauszniewski et al. (2013) | P11 Biofeedback control training | Feasibility | Pretest-posttest | U.S. (OH) | 20 | NA | Age *M* = 58 (*range* = 42-68); 100% female; 50% Black, 50% White; 50% less than college, 50% college -degreed | Any caregiver | Age ranged 6 months – 18 years |
| S20 Zauszniewski et al. (2014a) | P12 Resourcefulness training (RT) | Pilot | Quasi-experiment | U.S. (OH) | 40 | 40 | Age *M* = 58 (*SD* = 9); 100% female; 9% less than high school, 36% high school/GED, 55% some college or above | Any caregiver | Age ranged 6 months – 18 years |
| S21 Zauszniewski et al. (2014b) | P12 Resourcefulness training (RT) | Pilot | Quasi-experiment | U.S. (OH) | 40 (expression with RT) | 41 (expression only)  21 (control) | Age *M* = 58 (*range* = 40-82); 100% female; 60% Black, 32% White, 2% Asian, 1% American Indian; 11% less than high school, 33% high school/GED, 56% some college or above | Any caregiver | Age *M* = 9 |
| S22 Zauszniewski and Musil (2014) | P11 Biofeedback control training; P12 RT | Pilot | Quasi-experiment | U.S. (OH) | Biofeedback= 20; Resourcefulness = 20 | 20 | Age *M* = 57 (*range* = 40-82); 100% female; 54% Black, 38% White, 8% Asian, Hispanic, or American Indian; 34% less than high school, 51% high school graduate/ some college, 15% college-degreed | Any caregiver | Age ranged 6 months – 18 years |
| S23 Musil et al. (2015) | P12 Resourcefulness training | Feasibility | Pretest-posttest | U.S. (OH) | 12 | NA | Age *M* = 59.2 (*range* = 39-77); 100% female; 42% White, 17% Hispanic; 8% some high school, 8% high school graduate, 75% some college or above | Primary caregiver | 1-3 GC; Age ranged 6 months – 18 years |
| S24 Zauszniewski et al. (2017) | P12 Resourcefulness training | Feasibility | Pretest-posttest | U.S. (OH) | 20 | NA | Age *M* = 58 (*range* = 42-68); 100% female; 50% White, 50% Black; 60% less than college, 40% college graduate | Primary caregiver | Age ranged 6 months – 18 years |
| S25 N’zi et al. (2016) | P13 Child Directed Interaction Training (CDIT) / Parent-Child Interaction Therapy (PCIT) | Feasibility | RCT | U.S. (FL) | 7 | 7 | Age *M* = 56.5 (*range* = 45-73); 100% female; 64% White, 22% Black, 7% Hispanic, and 7% biracial; 7% less than high school, 7% high school graduate, 86% some college or above | Kinship foster caregiver | Age *M* = 5.2 (*range* = 3 months – 7.5 years); Having difficult behavior problems and a history of child maltreatment |
| S26 Young & Sharpe (2016) | P14 Intergenerational Physical Activity Intervention | Feasibility | Pretest-posttest | U.S. (FL) | 12 | NA | Age *M* = 32.3 (*SD* = 6.8); 100% female; 75% Black; 83% high school graduate | Any caregiver | 1.9 GC enrolled; *M* = 11.1 (*SD* = 3.7) |
| S27 Yancura et al. (2017) | P15 GRANDCares Project (Adapted from Powerful tools for Caregivers) | Feasibility | Pretest-posttest | U.S. (HI) | 9 | NA | Age *M* =65 (range = 51-73); 89% female; 67% Native Hawaiian, 11% Pacific Islander, 11% White, 11% Asian | Custodial caregiver | 3.25 GC; Aged range from 9months – 17 years |
| S28 Foli et al. (2018) | P16 Trauma-Informed Parenting Classes – Resource Parent Curriculum (RPC) | Feasibility | Pretest-posttest | U.S. (IN) | 16 | NA | Age *M* = 58.7 (*SD* = 7.5); 75% female; 100% White; 38% high school graduate/GED, 62% some college/ vocational/ technical | Primary/custodial caregiver | 1.4 GC; *M* = 6.8 (*SD* = 3.7) |
| S29 Zhang et al. (2018) | P17 Conditional cash transfer (CCT) program | Efficacy | RCT | China (Rural Hunnan) | 255 | 263 | Age *M* = 55.2 (*SD* = 12.3); 66% female; 63% Han Chinese, 37% ethnic minorities; 30% no education, 48% some elementary school, 22% some middle/ high school | Primary caregiver for left behind children | Age ranged 3-5 |
| S30 Montoro-Rodriguez and Hayslip (2019) | P18 Demonstration Project (Goal-Setting and Communications Skills Program) | Feasibility | Pretest-posttest | U.S. (TX ) | 16 | NA | Age *M* = 59 (*SD* = 5.4); 66% female; 63% Black, 38% White; 50% high school graduate, 50% some college | Custodial caregiver | Aged 18 below |
| S31 Hayslip et al. (2021) | P18 Demonstration Project (Goal-Setting and Communications Skills Program) | Pilot | RCT | U.S. (NC) | 34 | 16 | Age *M* = 59.1 (*SD* = 10.0); 98% female; 60% Black, 35% Hispanic, 4% White, 1% Asian; 40% high school graduate, 60% some college | Primary/custodial caregiver | 2 GC (*SD* = 1.4); aged 18 below |
| S32 Montoro-Rodriguez et al. (2021) | P18 Demonstration Project (Goal-Setting and Communications Skills Program) | Pilot | RCT | U.S. (NC) | 34 | 16 | Age *M* = 59.1 (*SD* = 10.0); 98% female; 60% Black, 35% Hispanic, 4% White, 1% Asian; 40% high school graduate, 60% some college | Primary/custodial caregiver | 2 GC (*SD* = 1.4); *M* = 10 (*SD* = 4.8) |
| S33 Xie et al. (2019) | P19 Active and Healthy Grandchildren | Feasibility | Pretest-posttest | U.S. (CA) | 12 | NA | 92% age > 60; 75% female; 88% Latinos of Mexican origin | Supplementary caregiver | Age ranged 2-12 |
| S34 Zakirova‑Engstrand et al. (2021) | P20 Psychoeducational intervention about autism spectrum disorder (ASD) | Feasibility | Pretest-posttest | Sweden (Stockholm) | 114 | NA | 57% age > 65; 64% female; 35% upper high school, 56% some college | Non-custodial caregiver for preschool-aged GC with ASD | 92% 1 GC with ASD; *M* = 4.1 (*Range* = 2-6, *SD* = 1.0) |
| S35 Fox et al. (2022) | P21 Powerful Tools for Caregivers – Grandfamilies | Feasibility | Pretest-posttest | U.S. (CO, HI) | 149 | NA | Age *M* = 62.0 (*SD* = 8.4); 79% female; 57% White, 15% Native Hawaiian or Pacific Islander, 10% Hispanic, 8% Asian, 7% American Indian, 2% Black; 28% high school graduate or less, 67% some college or above | Primary caregiver | Aged 18 below |

**Appendix 1: Search Strategy**

**PsycInfo (Ovid)**

1990 to January Week 4 2022

**Final 20220130**

*n* = 1083 2022 01 30

1. exp Grandparents/

2. (grandparent* or grandmother* or grandfather* or grandma* or grandpa*).mp.

3. (intergenerational).tw.

4. ("grandparent* raising" or "grandmother raising" or "grandfather* raising").tw.

5. (custody or custodial).tw.

6. exp Caregivers/

7. exp Coparenting/ or exp Child Custody/

8. exp Childrearing Practices/

9. exp Child Care/ or (babysit* or "baby sit*").tw.

10. casual.tw.

11. or/5-10

12. 1 or 2 or 3

13. 11 and 12

14. 4 or 13

15. limit 14 to treatment & prevention

16. limit 14 to ("0300 clinical trial" or 2100 treatment outcome)

17. exp treatment effectiveness evaluation/

18. (outcome* or effective* or efficacy or improv* or cohort* or trial* or rct or quasi- experiment* or compar* or control or waitlist* or pilot*).tw.

19. 14 and (17 or 18)

20. 15 or 16 or 19

21. limit 20 to (peer review journal and English)

22. limit 21 to yr="1990 -Current"

**Medline (Ovid)**

1990 to January Week 4 2022

**Final 20220130**

*n* = 448 2022 01 30

1. exp Grandparents/

2. (grandparent* or grandmother* or grandfather* or grandma* or grandpa*).mp.

3. (intergenerational).tw.

4. ("grandparent* raising" or "grandmother raising" or "grandfather* raising").tw.

5. (custody or custodial).tw.

6. exp Caregivers/

7. exp Child Custody/

8. exp Child Rearing/

9. exp Child Care/ or (babysit* or "baby sit*").tw.

10. casual.tw.

11. or/5-10

12. 1 or 2 or 3

13. 11 and 12

14. 4 or 13

15. Publication type: clinical trial, all or controlled clinical trial

16. exp treatment outcome/

17. (outcome* or effective* or efficacy or improv* or cohort* or trial* or rct or quasi-experiment* or compar* or control or waitlist* or pilot*).tw.

18. 14 and (16 or 17)

19. 18 or 15

20. limit 19 to English

21. limit 20 to yr="1990 -Current"

**CINAHL**

*n* =398

**Final 20220130**

S1 (MH "Grandparents")

S2 AB(( grandparent* or grandmother* or grandfather* ) AND ( custod* or raising or rearing or childcare or "child care" or grandfamil* or babysit* or "baby sit*" or casual))

S3 (MH "Treatment Outcomes")

S4 AB(outcome* or effective* or efficacy or improv* or cohort* or trial* or rct or quasi-experiment* or compar* or control or waitlist* or pilot*)

S5 S1 OR S2

S6 S3 OR S4

S7 S5 AND S6

S8 S5 AND S6 – English

S9 S5 AND S6 – Peer reviewed

S10 S5 AND S6 – 1990-2022

S11 S10 – Exclude Medline

**Scopus**

*n* = 213

**Final 20220130**

( TITLE-ABS-KEY (custod* W/3 ( grandparent* OR grandmother* OR grandfather* ) ) OR TITLE-ABS-KEY ( ( grandparent* OR grandmother* OR grandfather* ) W/3 raising ) OR TITLE-ABS-KEY ( grandfamil* ) OR TITLE-ABS-KEY ((babysit* or "baby sit*" or casual ) W/3 (grandparent* OR grandmother* OR grandfather*) ) )AND ( TITLE-ABS-KEY ( outcome* OR effective* OR efficacy OR improv* OR cohort* OR trial* OR rct OR quasi-experiment* OR compar* OR control OR waitlist* OR pilot* ) )

( LIMIT-TO ( SRCTYPE , "j" ) AND English )

**Appendix 2: National Institutes of Health (NIH) Quality Assessment tool**

**Controlled intervention study***

1. Was the study described as randomized, a randomized trial, a randomized clinical trial, or an RCT?

2. Was the method of randomization adequate (i.e., use of randomly generated assignment)?

3. Was the treatment allocation concealed (so that assignments could not be predicted)?

4. Were study participants and providers blinded to treatment group assignment?

5. Were the people assessing the outcomes blinded to the participants' group assignments?

6. Were the groups similar at baseline on important characteristics that could affect outcomes (e.g., demographics, risk factors, co-morbid conditions)?

7. Was the overall drop-out rate from the study at endpoint 20% or lower of the number allocated to treatment?

8. Was the differential drop-out rate (between treatment groups) at endpoint 15 percentage points or lower?

9. Was there high adherence to the intervention protocols for each treatment group?

10. Were other interventions avoided or similar in the groups (e.g., similar background treatments)?

11. Were outcomes assessed using valid and reliable measures, implemented consistently across all study participants?

12. Did the authors report that the sample size was sufficiently large to be able to detect a difference in the main outcome between groups with at least 80% power?

13. Were outcomes reported or subgroups analyzed prespecified (i.e., identified before analyses were conducted)?

14. Were all randomized participants analyzed in the group to which they were originally assigned, i.e., did they use an intention-to-treat analysis?

*For quasi-experimental designs, items 2-5 are removed, while item 1 is changed to “Is it clear in the study what is the ‘cause’ and what is the ‘effect’ (i.e. there is no confusion about which variable comes first)?”

**Pretest-posttest studies with no control group**

1. Was the study question or objective clearly stated?

2. Were eligibility/selection criteria for the study population prespecified and clearly described?

3. Were the participants in the study representative of those who would be eligible for the test/service/intervention in the general or clinical population of interest?

4. Were all eligible participants that met the prespecified entry criteria enrolled?

5. Was the sample size sufficiently large to provide confidence in the findings?

6. Was the test/service/intervention clearly described and delivered consistently across the study population?

7. Were the outcome measures prespecified, clearly defined, valid, reliable, and assessed consistently across all study participants?

8. Were the people assessing the outcomes blinded to the participants' exposures/interventions?

9. Was the loss to follow-up after baseline 20% or less? Were those lost to follow-up accounted for in the analysis?

10. Did the statistical methods examine changes in outcome measures from before to after the intervention? Were statistical tests done that provided p values for the pre-to-post changes?

11. Were outcome measures of interest taken multiple times before the intervention and multiple times after the intervention (i.e., did they use an interrupted time-series design)?

12. If the intervention was conducted at a group level (e.g., a whole hospital, a community, etc.) did the statistical analysis take into account the use of individual-level data to determine effects at the group level?
